# SJA, the dream became real

**DOI:** 10.4103/1658-354X.62605

**Published:** 2010

**Authors:** Abdulhamid Al-Saeed

**Affiliations:** Editor-in-Chief Saudi Journal Anaesthesia E-mail: editor@saudija.org


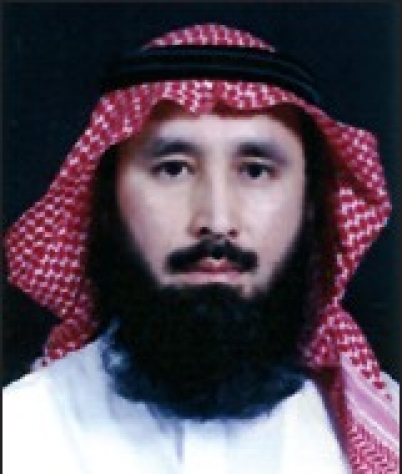


Saudi Journal of Anaesthesia (SJA) is the official Journal of the Saudi Anaesthetic Association (SAA). Since its establishment (in 2006), the SJA showed rapid and great progress. With the full support of his Excellency the President of King Saud University, Professor Abdullah Al Othman, we have carried out these new changes. The previous issue of the journal, in its new look, has been published by a well-recognized and established International publisher. In the last year you may have noticed that the SJA has been published online and this has given more strength to the journal, which is now on the path to international indexing. Since the SJA has come online, the number of articles is increasing and frankly the editorial board of the journal is paying more attention and working very hard to run the reviewing process in a closed loop module. Actually, the reviewing process takes time and effort, and this is cheerfully performed by the well-organized editorial board.

Till last year we were publishing two issues per year; from 2010 we are planning to publish three issues in a year. Also, we are working on special issues, which would include some key articles from anesthesia conferences held in the kingdom, besides other special issues on certain pre-specified topics. Our journal has been accepted for promotion by all the universities in the Kingdom and once it is indexed in PubMed, all the articles published previously will be indexed as well.

As Editor-in-Chief, I would like to thank all the members on the editorial board of the SJA and the consulting editors for the precious time they have spent in the management of both the printed as well as the online versions of SJA. Also, I would like to encourage our colleagues to send their articles to the journal office as online submissions, and as you might have noticed, the reviewing process does not take much time for a final decision to be taken.

The aim of the editorial board is to promote knowledge and research in anesthesia practice, besides bringing to the attention of the anesthesia community, all the new and forthcoming events, as a possible contribution.

We will be happy to receive your comments and suggestions, for a better performance and for improving the status of SJA, in order to reach our goal of being indexed internationally in the near future.

